# Phantom criteria for qualification of brain FDG and amyloid PET across different cameras

**DOI:** 10.1186/s40658-016-0159-y

**Published:** 2016-10-06

**Authors:** Yasuhiko Ikari, Go Akamatsu, Tomoyuki Nishio, Kenji Ishii, Kengo Ito, Takeshi Iwatsubo, Michio Senda

**Affiliations:** 1 Division of Molecular Imaging, Institute of Biomedical Research and Innovation, 2-2, Minatojima-Minamimachi, Chuo-ku, Kobe, 650-0047 Japan; 2 Research Team for Neuroimaging, Tokyo Metropolitan Institute of Gerontology, Tokyo, Japan; 3 Department of Clinical and Experimental Neuroimaging, National Center for Geriatrics and Gerontology, Obu, Japan; 4 Department of Neuropathology, Graduate School of Medicine, The University of Tokyo, Tokyo, Japan

**Keywords:** Brain FDG-PET, Amyloid PET, Image quality, Multicenter study, Standardization

## Abstract

**Background:**

While fluorodeoxyglucose (FDG) and amyloid PET is valuable for patient management, research, and clinical trial of therapeutics on Alzheimer’s disease, the specific details of the PET scanning method including the PET camera model type influence the image quality, which may further affect the interpretation of images and quantitative capabilities. To make multicenter PET data reliable and to establish PET scanning as a universal diagnostic technique and a verified biomarker, we have proposed phantom test procedures and criteria for optimizing image quality across different PET cameras.

**Results:**

As the method, four physical parameters (resolution, gray-white contrast, uniformity, and image noise) were selected as essential to image quality for brain FDG and amyloid PET and were measured with a Hoffman 3D brain phantom and a uniform cylindrical phantom on a total of 12 currently used PET models. The phantom radioactivity and acquisition time were determined based on the standard scanning protocol for each PET drug (FDG, ^11^C-PiB, ^18^F-florbetapir, and ^18^F-flutemetamol). Reconstruction parameters were either determined based on the methods adopted in ADNI, J-ADNI, and other research and clinical trials or optimized based on measured phantom image parameters under various reconstruction conditions.

As the result, phantom test criteria were proposed as follows: (i) 8 mm FWHM or better resolution and (ii) gray/white %contrast ≥55 % with the Hoffman 3D brain phantom and (iii) SD of 51 small region of interests (ROIs) ≤0.0249 (equivalent to 5 % variation) for uniformity and (iv) image noise (SD/mean) ≤15 % for a large ROI with the uniform cylindrical phantom. These criteria provided image quality conforming to those multicenter clinical studies and were also achievable with most of the PET cameras that are currently used.

**Conclusions:**

The proposed phantom test criteria facilitate standardization and qualification of brain FDG and amyloid PET images and deserve further evaluation by future multicenter clinical studies.

## Background

Brain PET imaging with fluorodeoxyglucose (FDG) and amyloid agents is promising for early and differential diagnosis of Alzheimer’s disease (AD) and is valuable for clinical research as well as for clinical trials of therapeutics [1–4].

However, PET image quality depends on the PET camera model and the specific reconstruction and acquisition details including injected activity, scan time, and reconstruction parameters, even if the radioactivity distribution is the same [5]. The image quality may affect image interpretation, quantitative capabilities, and even diagnostic capabilities, which makes it a challenge to acquire reliable data in a multicenter clinical study. To make PET a universal tool for research and therapeutic clinical trials as well as for patient management, the specific details of the scanning methods used should be “optimized” so that images of equivalent quality, both visually and quantitatively, can be obtained across different PET camera models.

In a well-controlled multicenter clinical research using PET on AD, such as Alzheimer’s Disease Neuroimaging Initiative (ADNI) [6], ADNI2 [7], and J-ADNI [8], and in industry-sponsored clinical trials [9, 10] on amyloid PET diagnostics or on therapeutics using brain FDG and amyloid PET, the PET QC manager has examined and qualified the PET cameras of each participating PET center based on phantom data. PET scanning details such as the reconstruction parameters are often determined during the qualification process so that images satisfying certain criteria can be obtained with each PET camera. However, no universally accepted phantom procedures and criteria have been published by academic societies. The details of the PET camera qualification procedures and criteria in industry-sponsored clinical trials are usually not open to the public.

In this work, we are proposing phantom procedures and criteria for qualification across different PET cameras to be used for brain FDG and amyloid PET imaging in multicenter studies. For that purpose, we first defined the elements of quality that are essential for brain FDG and amyloid PET images as physical parameters that are measurable in phantom experiments. Then, we examined the available details of PET scanning methods adopted in multicenter studies such as ADNI and J-ADNI and measured the physical parameters used with the phantoms to determine the “criteria,” based on which different PET cameras could be optimized. We also measured the physical parameters under various scanning conditions on a large number of PET camera models currently used in Japan to confirm that the criteria could be achieved by most of the currently used PET cameras under appropriate scanning conditions.

In terms of amyloid PET drugs, we dealt with ^11^C-PiB, ^18^F-florbetapir, and ^18^F-flutemetamol, because ^11^C-PiB has been used as a standard PET drug for research and the latter two ^18^F-labeled PET drugs are approved in many countries. ^18^F-florbetaben, which is also approved in many countries, was not dealt with in the present work because the PET camera used in the multicenter study for the efficacy of the PET drug was not available to us for the phantom experiments. However, the standard injection activity, scan time, % brain uptake, and other necessary information are provided in the “Discussion” section so that the readers can plan phantom experiments to evaluate a PET camera for ^18^F-florbetaben PET imaging.

## Methods

### Essential image quality for brain FDG PET

Detection and evaluation of localized hypometabolism is essential for the interpretation of FDG-PET images in research and clinical diagnosis regarding Alzheimer’s disease (AD) and other neurodegenerative disorders. AD is known to present a so-called AD pattern characterized by reduced FDG uptake in the temporoparietal cortex and in the posterior cingulate and precuneus, and other neurodegenerative disorders present other hypometabolic patterns [1]. Since FDG accumulates high in the cerebral cortex, PET images that have sufficient resolution provide structural information and help identification of lesion localization. The ring-shaped area along the contour of the brain on transaxial slices is called the cortical rim, which is actually a mixture of gray and white matter tissues interlacing each other. The apparent FDG uptake in the cortical rim reflects the proportion of gray matter tissue and is reduced if cortical atrophy occurs [11]. Therefore, poor resolution may make it difficult to distinguish pathological tissue hypometabolism from apparently decreased uptake due to atrophy.

In addition to the visual interpretation of FDG-PET images, the so-called statistical image analysis such as 3D-SSP is often used, in which the subject brain image is spatially normalized into a template and the relative regional uptake is compared voxel by voxel with the normal database to generate a z-map or t-map of significant hypometabolism [12, 13]. The z-map is either visually interpreted itself or further processed to generate a “score” representing the likelihood of the AD pattern [14, 15].

Therefore, FDG-PET images should have sufficiently high resolution and contrast together with sufficiently low noise to detect mild hypometabolism visually and quantitatively. Furthermore, image uniformity is also important because regional FDG uptake is evaluated as a relatively decreased activity in comparison with other areas both visually and quantitatively.

### Essential image quality for brain amyloid PET

It is essential to detect and gauge abnormal cortical uptake in amyloid PET imaging as it reflects pathological deposition of amyloid beta plaque. A positive scan is characterized by such abnormal uptake and is found in most AD patients and in some cognitively normal elderly subjects, while a negative scan is characterized by the absence of such abnormal cortical uptake [16]. There are a number of PET drugs used for amyloid PET imaging, including ^11^C-PiB, ^18^F-florbetapir, ^18^F-florbetaben, and ^18^F-flutemetamol, but all of them accumulate non-specifically in the white matter [17–20]. Therefore, it is necessary to detect mild cortical uptake adjacent to the non-specific uptake in the white matter, which requires sufficiently high resolution and contrast as well as low noise. In the case of cortical atrophy, this may often be a challenge.

Quantitative analysis of amyloid PET images is used as an adjunct to the visual interpretation as well as for the evaluation of disease progression and the monitoring of treatment. The ratio of cortex to cerebellum or pons as a reference region (SUVR) is the most frequently used indicator [19, 21–23]. The quantitative measurement of the regional cortical uptake is influenced by a partial volume effect due to limited resolution, in which both spill-in from the white matter and spill-out into the CSF space occur [24]. Noise degrades the quantitative precision. Furthermore, quantitative capability is essential for the reference region. Therefore, uniformity within the field of view is also important in amyloid PET in addition to resolution, gray-white contrast, and noise.

### Phantoms and physical parameters measured

Based on the above insights, we decided on the following four physical quality parameters for the phantom criteria and the phantoms to be used for measurement.

(i) Resolution: Hoffman 3D brain phantom
(ii) Gray-white contrast: Hoffman 3D brain phantom
(iii) Uniformity: uniform cylindrical phantom
(iv) Image noise: uniform cylindrical phantom

We chose the Hoffman 3D brain phantom (Data Spectrum Corporation, Durham North Carolina) because it is commercially available with a unique specification and because it simulates gray-matter and white-matter structures with 4:1 activity concentration, which is ideal for predicting the image quality of FDG-PET. A gray-white ratio of 4:1 is too high when it comes to detecting mild cortical uptake in amyloid PET, but it can still provide indicators of resolution and contrast and is considered instrumental for predicting the image quality of amyloid PET. The uniform cylindrical phantom has an inner diameter of 16 cm and an inner length of 30 cm and is also commercially available.

Table 1 presents the phantom radioactivity and the scan time (data acquisition time) adopted in the proposed phantom procedures to simulate a standard PET scan with each PET drug. They were derived from the standard injection activity, physical decay during the accumulation time, average brain uptake, and standard scan time for each PET drug, based on the following considerations.

**Table 1:** Phantom activity at start of scan and the interval to be extracted from list mode phantom data for each PET drug

	Hoffman phantom	Cylindrical phantom
Activity at scan start	20 MBq	40 MBq
FDG	1800 s	865 s
PiB	135 s	70 s
Florbetapir	710 s	350 s
Flutemetamol	255 s	180 s

Ideally, the phantom is to be filled with the amount of radioactivity that would exist in the brain at the start of the human PET scan, which is a function of injection activity, accumulation time (period between injection and start of emission scan, also called uptake time), and % brain uptake, and depends on the PET drug and protocol as well as on the pathological status of the subject. However, in view of efficiency and simplicity, we propose to determine a unique radioactivity value for each phantom regardless of the PET drug and the study protocol and to adjust the phantom scan time to match the activity-time product derived from the scanning protocol for each PET drug. As long as the injection activity is not too high for the count rate characteristics of the PET camera, as in most of the currently used PET cameras, the activity-time product determines the amount of available gamma ray counts. Practically, the phantom data are acquired in a long list mode so that the interval corresponding to each PET drug can be extracted and forwarded for image reconstruction. This allows phantom evaluation for two or more PET drugs in one experiment.

Table 2 presents the injection activity, accumulation time, and estimated brain activity at the start of scan for each PET drug that were used to derive the phantom experiment protocol proposed in this article. The injection activity is specified and standardized in most situations, and we adopted the values according to ADNI, ADNI2, J-ADNI, clinical trials of the PET drugs, and Japanese Society of Nuclear Medicine (JSNM) guidelines [7–10, 12, 25–27]. The accumulation time should be standardized as much as possible because it affects the distribution of radioactivity, and we followed the standard methods adopted by previous studies. On the other hand, the scan time (duration of emission scan) has been variable and may even be determined specifically for each camera within the same project by phantom experiments through the qualification process, depending on the camera sensitivity. We adopted the standard scan time values written in the JSNM guidelines, which were based on previous studies and clinical trials, but they may be changed depending on the actual scan time. The % brain uptake depends on the pathophysiology, and we adopted the average values for each PET drug from the literature or through personal communication with investigators. Detailed explanations for each PET drug are given below.

**Table 2:** Scanning protocols and assumed brain activity at scan start that are used to derive the phantom methods of Table 1

PET drug	Standard injection activity	Accumulation time	Standard scan time	Estimated brain activity at start of scan
FDG	185 MBq	30 min	30 min	20 MBq
PiB	555 MBq	50 min	20 min	3 MBq
Florbetapir	370 MBq	50 min	20 min	12 MBq
Flutemetamol	185 MBq	90 min	30 min	3 MBq

For FDG, we followed the protocol of ADNI and J-ADNI, in which injection activity was 185 MBq, accumulation time was 30 min, and scan time was 30 min [28].

Formerly, the accumulation time of a typical brain FDG-PET study ranged from 45 to 60 min, when the regional uptake reflects glucose metabolism based on the tracer kinetics [29], which is necessary for quantitative measurement of glucose metabolism. However, if the purpose is identification of hypometabolic pattern and differential diagnosis, then a shorter accumulation time is equally effective because the regional blood flow, which the earlier scan reflects more, parallels the regional metabolism in neurodegenerative disorders [30]. The % brain uptake of FDG at 30 min post-injection was assumed to be 13 %ID based on the time-%ID curve for the brain [29] leading to estimated brain activity of 20 MBq at 30 min post-injection, with decay taken into account. This phantom activity is comparable to the 0.5–0.6 mCi that was used in the camera qualification process for ADNI [5].

For ^11^C-PiB, we also followed the protocol of ADNI and J-ADNI, in which injection activity was 555 MBq, accumulation time was 50 min, and scan time was 20 min. The % brain uptake was assumed to be 3 % (0.53 %IRD) based on the brain time-%IRD curve in a previous report [31] (%IRD denotes % injected radioactive dose, which is %ID with decay), leading to an estimated brain activity of 3 MBq.

For ^18^F-florbetapir and ^18^F-flutemetamol, standard injection activity was 370 and 185 MBq, accumulation time was 50 and 90 min, and standard scan time was 20 and 30 min, respectively. Although the package insert of ^18^F-florbetapir describes 10 min as the scan time, we have adopted a scan time of 20 min according to ADNI2 [32] and other research protocols and the JSNM guidelines [26]. Similar situations were found for the scan time of ^18^F-flutemetamol, of which the package insert indicated 20 min, but we adopted a scan time of 30 min according to the clinical trial protocols [33] and the JSNM guidelines [26].

The % brain uptake was assumed to be 4.5 %ID and 3.0 %ID based on the dosimetry study for ^18^F-florbetapir [34] and ^18^F-flutemetamol [35], leading to estimated brain activity of 12 and 3 MBq, respectively.

Amyloid-positive subjects present higher cortical uptake than amyloid-negative subjects (around 1.5 to 2 times the cortical SUV, depending on the PET drug [12, 17, 22, 23, 36]). However, a unique phantom protocol was determined for each PET drug, because the intensity and extent of increased uptake is variable among subjects and because it is important to detect mild cortical uptake rather than strong extensive uptake.

### Phantom data acquisition

A Hoffman 3D brain phantom was filled with 20 MBq of ^18^F solution (FDG) at the start of scanning and scanned in a list mode or dynamic mode for 30 min together with a cylindrical phantom containing 80 MBq of ^18^F solution (FDG) placed on the bed 30 cm apart from the end of the phantom simulating the body activity. Data acquired during the “acquisition times” described in Table 1 were extracted from the list mode or dynamic mode data and reconstructed with specified or various parameters and post-filters. The scan of the uniform cylindrical phantom started when the activity decayed to 40 MBq and lasted for 30 min in a list or dynamic mode. Data acquired during the “acquisition times” described in Table 1 were extracted from the list mode or dynamic mode data and reconstructed with specified or various parameters and post-filters.

In the case of using a PET camera without list mode to acquire phantom data as the four PET drugs in Table 1, the Hoffman phantom data were acquired with a dynamic scan of four frames (135, 120, 455, and 1090 s). Averaged frames were provided for images to be evaluated. For example, the combination of the first (135 s) and second (120 s) frames is for flutemetamol (255 s).

To optimize the image reconstruction parameters, we vary the resolution and noise by changing iteration and subset combinations. In the case of particularly noisy images, we implemented Gaussian post-filters to control image noise, i.e., a gauss filter of 4 mm FWHM.

### Phantom image analysis

**Fig. 1:**
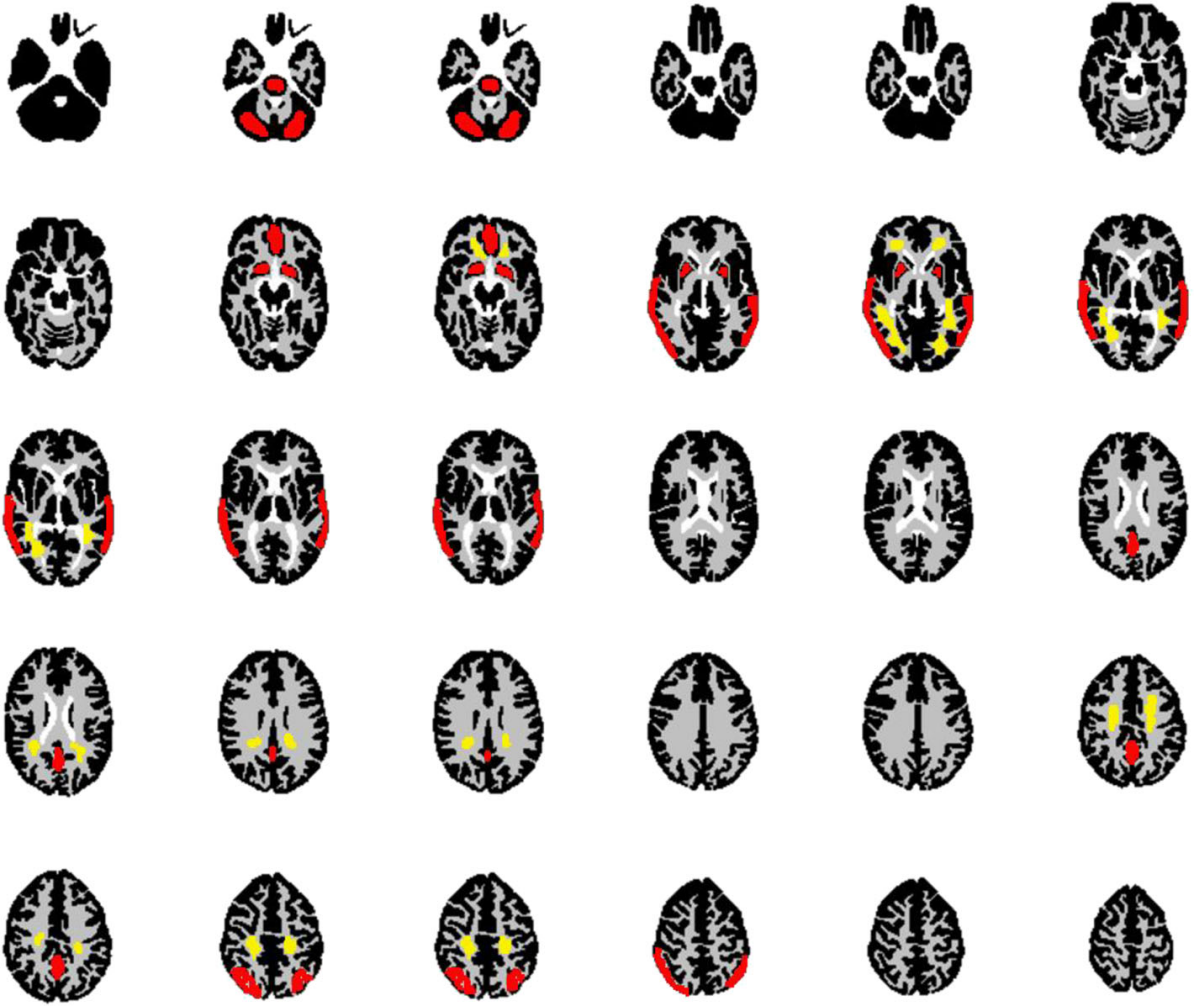
ROI template (*red* for gray matter, *yellow* for white matter) defined on the digital Hoffman phantom for evaluation of gray/white contrast

Spatial resolution and gray/white contrast were computed from the Hoffman phantom images in the following manner. Spatial resolution was estimated from visual similarity between the Hoffman phantom image and the digital phantom obtained from the vendor treated with a 3D Gaussian filter of various FWHMs [37]. To derive the gray/white contrast, the JSNM region of interest (ROI) templates were defined on the digital Hoffman phantom that would provide a true gray-to-white ratio of 4 and were applied to the phantom image co-registered to the digital phantom (Fig. 1) [26, 38]. The %contrast was calculated as follows:

$$ \%\mathrm{contrast}=\frac{\left({\mathrm{GM}}_p/{\mathrm{WM}}_p-1\right)}{\left({\mathrm{GM}}_d/{\mathrm{WM}}_d-1\right)}\times 100 $$

where GM_*p*_ and WM_*p*_ were the ROI activity of gray matter and white matter measured on the phantom PET image, respectively, and GM_*d*_ and WM_*d*_ were the ROI activity of gray matter and white matter on the digital phantom, respectively.

The JSNM ROI templates were provided from gray/white sections in the Hoffman Digital Phantom Image and scraped along boundary voxels in order to avoid partial volume effect.

Uniformity and noise were evaluated in the uniform cylindrical phantom images in the following manner. For uniformity evaluation, 17 circular ROIs of 500 mm^2^ (uROI) were placed on the central slice and on two other slices ±40 mm apart from the central slice, making a total of 51 uROIs. The SD_uROI mean_ was calculated as follows:

$$ {\mathrm{SD}}_{\mathrm{uROI}\ \mathrm{mean}}=\sqrt{\frac{1}{n}{\displaystyle \sum_{i=1}^n{\left(\frac{{\mathrm{uROI}}_{\mathrm{mean}}}{{\mathrm{uROI}}_{\mathrm{TOT}}}-1\right)}^2}} $$

where uROI_mean_ was the mean activity of each uROI, *n* = 51, and uROI_TOT_ was the average of the 51 uROI_mean_.

For noise evaluation, a large circular ROI of 130 mm in diameter (nROI) was placed on the central slice. The coefficient of variation (CV) was calculated as follows:

$$ \mathrm{C}\mathrm{V}=\frac{{\mathrm{SD}}_{\mathrm{nROI}}}{{\mathrm{nROI}}_{\mathrm{mean}}}\times 100\ \left[\%\right] $$

where SD_nROI_ was the standard deviation of the voxel values within the nROI and nROI_mean_ was the mean nROI activity.

### Phantom evaluation under previous clinical protocols

Details of PET scanning methods of previous multicenter clinical study projects that had been carried out using FDG, PiB, ^18^F-florbetapir, or ^18^F-flutemetamol were collected from the literature, presentations at scientific meetings, or through personal communications from the investigators and PET QC managers of those projects. As a result, information about the PET camera model, injection activity, accumulation time, scan time, and reconstruction conditions were obtained for ADNI, J-ADNI, and clinical trials on efficacy of ^18^F-labeled amyloid PET drugs.

The phantom images were acquired based on the procedures described above using the PET cameras of the same model under the same scanning protocols as were used in those previous clinical studies, including injection activity, accumulation time, scan time, reconstruction conditions, and post-filters. The physical quality parameters (spatial resolution, gray-white contrast, uniformity, and image noise) were measured on the phantom images.

### Phantom evaluation for currently used PET cameras

Phantom data were acquired on 19 PET cameras of 12 different models from 15 PET centers that participated in the J-ADNI2 project according to the procedures described above. The data for the four intervals corresponding to the four PET drugs described in Table 1 were extracted and reconstructed with various parameters and post-filters.

Table 4 shows the optimized reconstruction conditions that were selected and the physical parameters that were measured in this study. The detailed method for determining the optimized reconstruction parameters for an individual PET camera is reported by Akamatsu et al. [38].

### Determination of phantom criteria

The phantom criteria were proposed based on these data so that it conforms to the image quality and quantitative capability provided by ADNI, J-ADNI, and clinical trials and so that most PET camera models could meet the criteria by selecting appropriate reconstruction parameters.

## Results

Table 3 summarizes the scanning conditions including the reconstruction parameters for each PET camera model used in PET scans with FDG, PiB, ^18^F-florbetapir, and ^18^F-flutemetamol in ADNI, ADNI2, J-ADNI, and clinical trials on ^18^F-florbetapir and ^18^F-flutemetamol. The phantom data were obtained according to the corresponding protocol, and the physical parameters measured on the phantom images were also presented. The spatial resolution was 9 mm FWHM or better in all cases and 7 mm FWHM or better in most of them. The %contrast ranged from 51.0 to 65.5 and was inversely associated with the FWHM values. The uniformity (SD) was below 0.0249, and the image noise ranged from 2.7 to 20.5.

**Table 3:** PET camera models and protocols used in clinical studies and physical parameters measured with phantoms

PET agent	Vendor, model	Reconstruction parameters	Study	Spatial resolution (mm)	%contrast	Uniformity (SD)	Image noise (CV [%])
FDG	GE, Advance	FORE + OSEM, subset = 20, iteration = 4, *Z*-axis; none	J-ADNI^a^	7	61	0.0249	13.7
	Shimadzu, Eminence GM	HDE, FORE-DRAMA, filter cycle = 0, iteration = 4		7	55	0.0249	8.8
	Shimadzu, HeadtomeV	FORE + OSEM, subset = 16, iteration = 4, Ramp × BW cf = 8 o = 2		7	61.6	0.0200	9.6
	Shimadzu, HeadtomeV	FORE + OSEM, subset = 16, iteration = 4, Ramp x BW cf = 8 o = 2		7	65.5	0.0230	9.7
	SIEMENS, biograph Hi-Rez	FORE + OSEM, subset = 14, iteration = 4		7	64.4	0.0130	7.6
	SIEMENS, biograph Hi-Rez	FORE + OSEM, subset = 16, iteration = 4		7	63.1	0.0150	6.9
	SIEMENS, biograph truePoint	FORE + OSEM, subset = 14, iteration = 4		6	61.3	0.0100	7.1
	SIEMENS, biograph Hi-Rez	FORE + OSEM, subset = 16, iteration = 4, Gaussian (XxYxZ = 5.5 × 5.5 × 5.5)	ADNI^a^	*9*	*52.8*	0.0120	2.73
	SIEMENS, ECAT Accel	FORE + OSEM, subset = 16, iteration = 6, Gaussian (XxYxZ = 2.0 × 2.0 × 3.0)		7	*54.6*	0.0200	6.03
Florbetapir	GE, Discovery690	3D-iteration, subset = 16, iteration = 4, Gaussian (XxYxZ = 5 mm)	*1	7	56.9	0.0120	6.2
	GE, Discovery690	3D-iteration, subset = 18, iteration = 3, Gaussian (XxYxZ = 2 mm), PSF (+)	*2	6	58	0.0110	11.7
	SIEMENS, biograph Hi-Rez	FORE + OSEM, subset = 16, iteration = 4, Gaussian (XxYxZ = 5.5 × 5.5 × 5.5)	ADNI^a^	7	62.8	0.0120	11
	SIEMENS, ECAT Accel	FORE + OSEM, subset = 16, iteration = 6, Gaussian (XxYxZ = 2.0 × 2.0 × 3.0)		7	55.4	0.0200	10
Flutemetamol	GE, Discovery690	3D-iteration, subset = 32, iteration = 3, Gaussian (XxYxZ = 5 mm), TOF (+)	*3	6.5	55.7	0.0230	8.8
PiB	GE, Advance	FORE + OSEM, subset = 20, iteration = 4, *Z*-axis; none, Gaussian (XxYxZ = 4 mm)	J-ADNI^a^	7	58	0.0249	*17.9*
	Shimadzu, Eminence GM	HDE, FORE-DRAMA, filter cycle = 0, iteration = 4, Gaussian (XxYxZ = 4 mm)		8	*51*	0.0249	13.7
	Shimadzu, HeadtomeV	FORE + OSEM, subset = 16, iteration = 4, Ramp × BW cf = 8 o = 2, Gaussian (XxYxZ = 4 mm)		8	*53.7*	0.0200	*18.4*
	Shimadzu, HeadtomeV	FORE + OSEM, subset = 16, iteration = 4, Ramp × BW cf = 8 o = 2, Gaussian (XxYxZ = 4 mm)		7	57.7	0.0230	*16.1*
	SIEMENS, biograph Hi-Rez	FORE + OSEM, subset = 14, iteration = 4, Gaussian (XxYxZ = 4 mm)		8	59.5	0.0130	12.4
	SIEMENS, biograph Hi-Rez	FORE + OSEM, subset = 16, iteration = 4, Gaussian (XxYxZ = 4 mm)		8	58.3	0.0150	11.8
	SIEMENS, biograph truePoint	FORE + OSEM, subset = 14, iteration = 4, Gaussian (XxYxZ = 4 mm)		8	56.4	0.0100	10.9
	SIEMENS, biograph Hi-Rez	FORE + OSEM, subset = 16, iteration = 4, Gaussian (XxYxZ = 5.5 × 5.5 × 5.5)	ADNI^a^	*9*	*52.1*	0.0120	7.78
	SIEMENS, ECAT Accel	FORE + OSEM, subset = 16, iteration = 6, Gaussian (XxYxZ = 2.0 × 2.0 × 3.0)		*9*	*51.8*	0.0200	*20.49*

Italic numbers represent performances deviated from the proposed criteria of phantom test. For *1 and *2, injection activity, accumulation time, and scan time are 370 MBq, 50 min, and 10 min, respectively, in clinical trials with florbetapir. For *3, injection activity, accumulation time, and scan time are 185 MBq, 90 min, and 30 min, respectively, in clinical trial with flutemetamol. See text and cited literatures

^a^In ADNI and J-ADNI, injection activity, accumulation time, and scan time are 185 MBq, 30 min, and 30 min for FDG, 555 MBq, 50 min, and 20 min for PiB, 370 MBq, 50 min, and 20 min for florbetapir, respectively

**Fig. 2:**
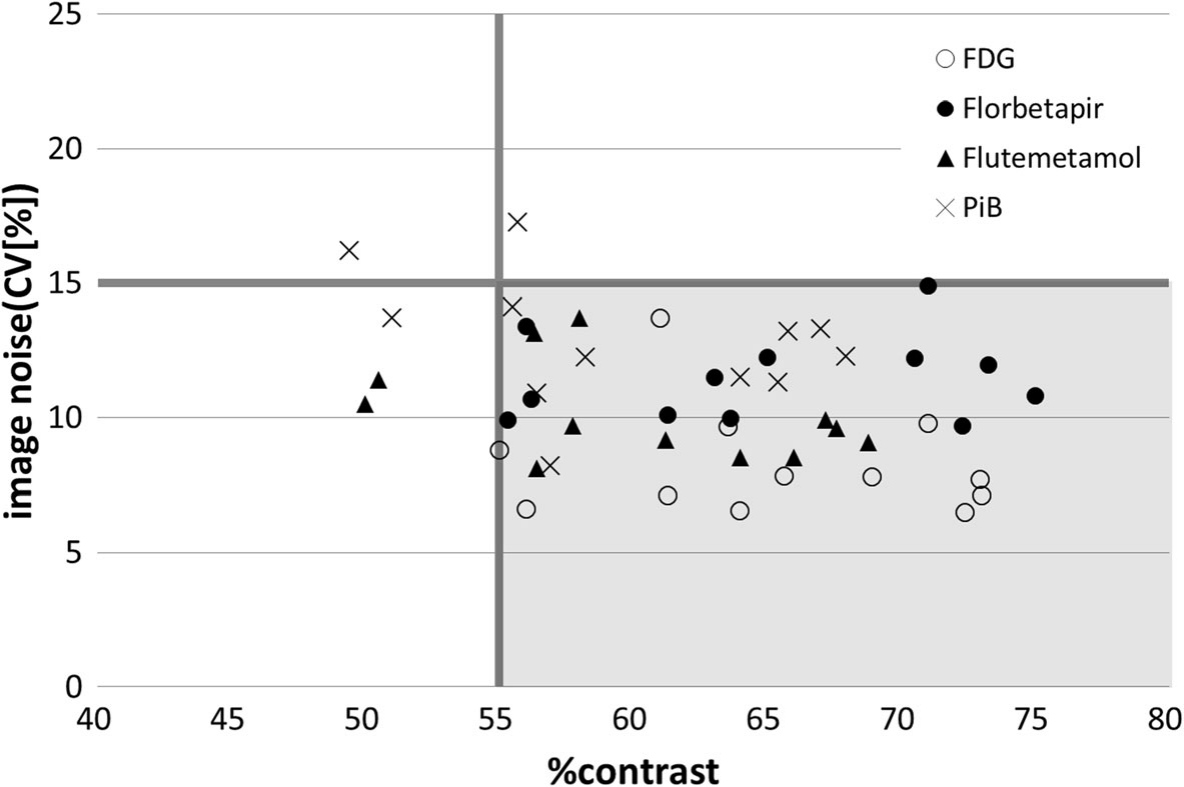
Scatter plots of %contrast and image noise (CV [%]) of phantom images reconstructed with optimized parameters for each camera and PET drug. Each point stands for each camera with adapted reconstruction parameter. Some camera needed to select parameters that were different from clinical settings. There was the trade-off between %contrast and image noise (CV [%])

**Fig. 3:**
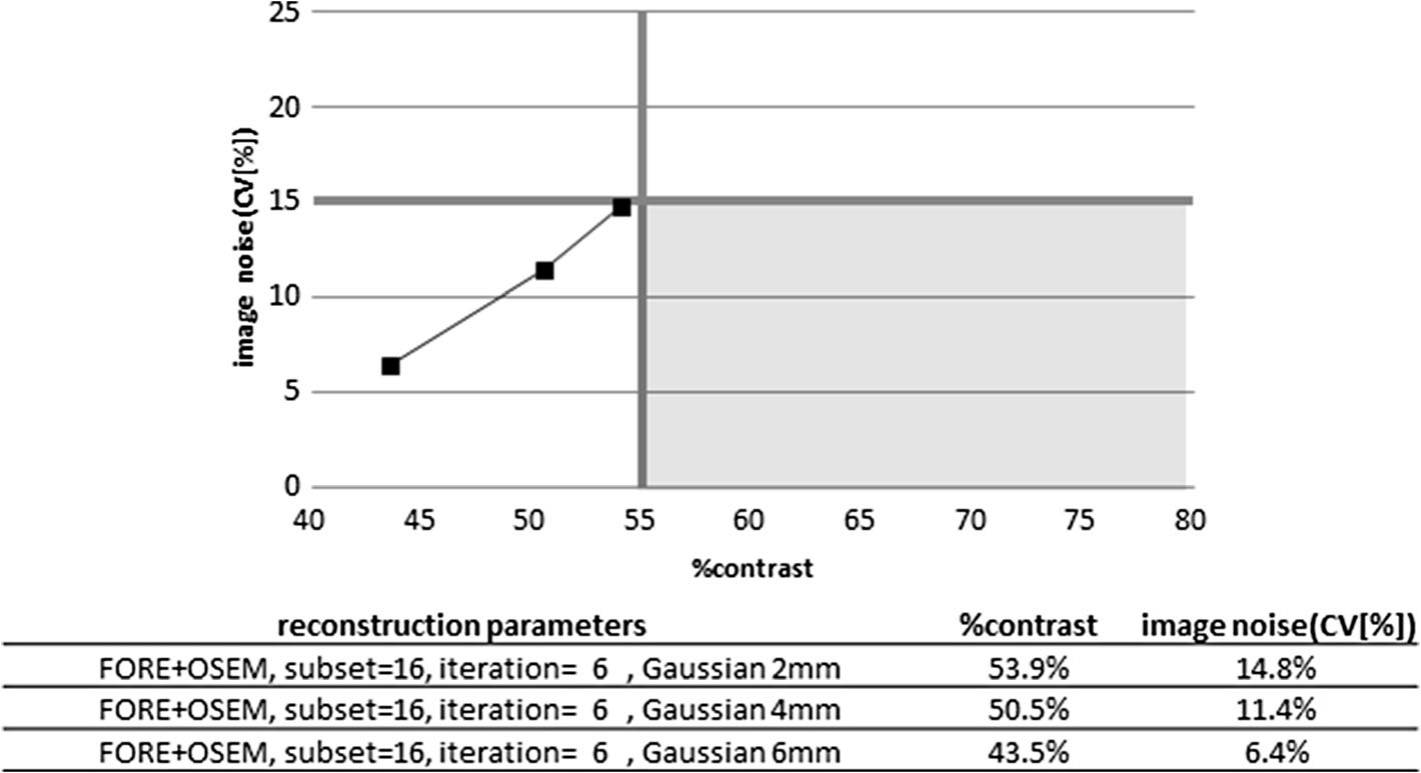
Relationship between %contrast and image noise (CV) with the reconstruction parameter (96 iterative updates: iteration = 6 and subset = 16) and different post-filters (2, 4, and 6 mm FWHM Gaussian filter) for an old PET camera model measured with the phantoms under flutemetamol condition. Ninety-six iterative updates and other iterative updates (80 and 128, data not shown) did not satisfy the criteria (*shaded region*)

Figure 2 plots the %contrast measured with the Hoffman phantom against the image noise (CV) measured with the cylindrical phantom for the 12 PET camera models that were acquired with the protocol for each PET drug and reconstructed with the conditions that were considered appropriate in terms the trade-off between %contrast and image noise. For most PET cameras, optimized reconstruction conditions were found that provided %contrast 55 % or higher and image noise (CV) 15 % or lower. However, in three PET camera models, which were rather old types, no reconstruction conditions provided %contrast and image noise within the above range under the phantom protocol for one or more PET drugs. Figure 3 depicts such a case, in which changing the reconstruction parameters (subjects and iterations) and post-filter resulted in a trade-off between %contrast and image noise and did not reach an image that satisfies both criteria.

Based on these results, we decided to propose “55 % or higher” as the criteria for the %contrast and “15 % or lower” as the criteria for CV. We also propose 8 mm FWHM as the criteria for the spatial resolution, because the spatial resolution was 8 mm FWHM or better whenever %contrast was 55 % or higher. As for uniformity (SD), we adopted 0.0249 or lower.

In contrast with Table 3 of values obtained from national projects and clinical trials, Table 4 summarizes the optimized image results provided by optimized reconstruction parameters for 12 PET cameras. Most PET cameras met the criteria except for some old ones. The spatial resolution was 8 mm FWHM or better, and the uniformity (SD) was below 0.0249 in all models. The SD of 0.0249 corresponds to 95 % of the uROI mean values within 5 % of the mean assuming normal distribution.

**Table 4:** Phantom image performances acquired with reconstruction parameters optimized for each PET camera and for each PET drug

Vendor, model	PET drug	Reconstruction parameters	Spatial resolution (mm)	%contrast (%)	Uniformity (SD)	Image noise (CV [%])
GE, Advance	FDG	FORE + OSEM, subset = 20, iteration = 4, *Z*-axis; none	7.0	61.0	0.0245	13.7
	Florbetapir	FORE + OSEM, subset = 20, iteration = 4, *Z*-axis; none, Gaussian 4 mm	7.0	56.2	0.0245	10.7
	Flutemetamol	FORE + OSEM, subset = 20, iteration = 4, *Z*-axis; none, Gaussian 4 mm	7.0	58.0	0.0245	13.7
	PiB	FORE + OSEM, subset = 20, iteration = 4, *Z*-axis; none, Gaussian 4.5 mm	7.0	55.5	0.0245	14.1
GE, Discovery 600^a^	FDG	3D-iteration, subset = 16, iteration = 5	5.3	72.9	0.0103	7.7
	Florbetapir	3D-iteration, subset = 16, iteration = 5	5.3	73.3	0.0103	12.0
	Flutemetamol	3D-iteration, subset = 16, iteration = 5, Gaussian (XxYxZ = 4 mm)	6.7	68.8	0.0103	9.1
	PiB	3D-iteration, subset = 16, iteration = 5, Gaussian (XxYxZ = 4 mm)	6.7	67.9	0.0103	12.3
GE, Discovery 690/710^a^	FDG	3D-iteration, subset = 16, iteration = 4	5.3	65.6	0.0107	7.8
	Florbetapir	3D-iteration, subset = 16, iteration = 4	5.3	65.0	0.0115	12.3
	Flutemetamol	3D-iteration, subset = 16, iteration = 4, Gaussian (XxYxZ = 4 mm)	6.3	61.2	0.0107	9.2
	PiB	3D-iteration, subset = 16, iteration = 4, Gaussian (XxYxZ = 5 mm)	6.2	56.9	0.0110	8.2
GE, Discovery ST Elite	FDG	3D-iteration, subset = 35, iteration = 2, *Z*-axis; standard	5.5	68.9	0.0140	7.8
	Florbetapir	3D-iteration, subset = 35, iteration = 2, *Z*-axis; standard	5.5	70.5	0.0140	12.2
	Flutemetamol	3D-iteration, subset = 35, iteration = 2, *Z*-axis; standard, Gaussian (XxYxZ = 4 mm)	6.0	67.2	0.0140	9.9
	PiB	3D-iteration, subset = 35, iteration = 2, *Z*-axis; standard, Gaussian (XxYxZ = 4 mm)	6.0	67.0	0.0140	13.3
GE, Discovery ST (upgraded for 3D-IR)	FDG	3D-iteration, subset = 21, iteration = 4, *Z*-axis; standard	6.0	73.0	0.0120	7.1
	Florbetapir	3D-iteration, subset = 21, iteration = 4, *Z*-axis; standard	6.0	75.0	0.0120	10.8
	Flutemetamol	3D-iteration, subset = 21, iteration = 4, *Z*-axis; standard, Gaussian (XxYxZ = 4 mm)	6.0	67.6	0.0120	9.6
	PiB	3D-iteration, subset = 21, iteration = 4, *Z*-axis; standard, Gaussian (XxYxZ = 4 mm)	6.0	65.8	0.0120	13.2
Shimadzu, HeadtomeV^b^	FDG	FORE + OSEM, subset = 16, iteration = 4, Ramp × BW cf = 8 o = 2	7.0	63.6	0.0215	9.7
	Florbetapir	FORE + OSEM, subset = 16, iteration = 4, Ramp × BW cf = 8 o = 2	7.0	63.1	0.0215	11.5
	Flutemetamol	FORE + OSEM, subset = 16, iteration = 4, Ramp × BW cf = 8 o = 2, Gaussian (XxYxZ = 4 mm)	7.5	56.3	0.0215	13.2
	PiB	FORE + OSEM, subset = 16, iteration = 4, Ramp × BW cf = 8 o = 2, Gaussian (XxYxZ = 4 mm)	7.5	55.7	0.0215	*17.3*
Shimadzu, Eminence BX	FDG	HDE, FORE-DRAMA, filter cycle = 0, iteration = 4	6.0	72.4	0.0180	6.5
	Florbetapir	HDE, FORE-DRAMA, filter cycle = 0, iteration = 4	6.0	72.3	0.0180	9.7
	Flutemetamol	HDE, FORE-DRAMA, filter cycle = 0, iteration = 4, Gaussian (XxYxZ = 4 mm)	7.0	66.0	0.0180	8.5
	PiB	HDE, FORE-DRAMA, filter cycle = 0, iteration = 4, Gaussian (XxYxZ = 4 mm)	7.0	65.4	0.0180	11.3
Shimadzu, Eminence GM	FDG	HDE, FORE-DRAMA, filter cycle = 0, iteration = 4	7.0	55.0	0.0249	8.8
	Florbetapir	HDE, FORE-DRAMA, filter cycle = 0, iteration = 4	7.0	56.0	0.0249	13.4
	Flutemetamol	HDE, FORE-DRAMA, filter cycle = 0, iteration = 4, Gaussian (XxYxZ = 4 mm)	8.0	*50.0*	0.0249	10.5
	PiB	HDE, FORE-DRAMA, filter cycle = 0, iteration = 4, Gaussian (XxYxZ = 4 mm)	8.0	*51.0*	0.0249	13.7
SIEMENS, biograph Hi-Rez^a^	FDG	FORE + OSEM, subset = 14 (16), iteration = 4	7.0	64.0	0.0140	6.5
	Florbetapir	FORE + OSEM, subset = 14 (16), iteration = 4	7.0	63.6	0.0140	10.0
	Flutemetamol	FORE + OSEM, subset = 14 (16), iteration = 4, Gaussian (XxYxZ = 4 mm)	8.0	57.8	0.0135	9.7
	PiB	FORE + OSEM, subset = 14 (16), iteration = 4, Gaussian (XxYxZ = 4 mm)	8.0	58.2	0.0140	12.2
SIEMENS, biograph mCT-X 3R	FDG	3D-iterative, subset = 12, iteration = 4	6.0	71.0	0.0150	9.8
	Florbetapir	3D-iterative, subset = 12, iteration = 4	6.0	71.0	0.0150	14.9
	Flutemetamol	3D-iterative, subset = 12, iteration = 4, Gaussian (XxYxZ = 4 mm)	7.0	64.0	0.0150	8.5
	PiB	3D-iterative, subset = 12, iteration = 4, Gaussian (XxYxZ = 4 mm)	7.0	64.0	0.0150	11.5
SIEMENS, biograph truePoint	FDG	FORE + OSEM, subset = 14, iteration = 4	6.0	61.3	0.0100	7.1
	Florbetapir	FORE + OSEM, subset = 14, iteration = 4	6.0	61.3	0.0100	10.1
	Flutemetamol	FORE + OSEM, subset = 14, iteration = 4, Gaussian (XxYxZ = 4 mm)	8.0	56.4	0.0100	8.1
	PiB	FORE + OSEM, subset = 14, iteration = 4, Gaussian (XxYxZ = 4 mm)	8.0	56.4	0.0100	10.9
SIEMENS, ECAT Accel	FDG	FORE + OSEM, subset = 16, iteration = 6	7.0	56.0	0.0210	6.6
	Florbetapir	FORE + OSEM, subset = 16, iteration = 6	7.0	55.3	0.0210	9.9
	Flutemetamol	FORE + OSEM, subset = 16, iteration = 6, Gaussian (XxYxZ = 4 mm)	8.0	*50.5*	0.0210	11.4
	PiB	FORE + OSEM, subset = 16, iteration = 6, Gaussian (XxYxZ = 4 mm)	8.0	*49.4*	0.0210	*16.2*

Italic numbers represent performances deviated from the proposed criteria of phantom test for the specific PET drug condition

^a^The parameters are the mean values of three cameras of the same model

^b^The parameters are the mean values of two cameras of the same model

## Discussion

To make multicenter PET data reliable and to establish the PET scan as a universal tool for brain studies, we have proposed phantom test procedures and criteria to optimize the image quality across different PET cameras.

It should be emphasized that no theoretical absolutes exist as reference values for those physical parameters of the phantom test. If the reference values are set at a high level, then the PET images acquired in the multicenter study will be of higher quality, which may possibly lead to a result demonstrating higher diagnostic capability of the PET imaging for the study population. However, only a few PET camera models will meet the criteria and can be used for the study project, which may reduce the number of participating PET centers and, accordingly, limit the number of study cases. If a new PET drug is approved by the regulatory authorities based on the multicenter study data, and if the phantom criteria become a requirement for the PET camera to be used, the PET scan may not be readily available to the public. On the other hand, if the reference values are set at a low level, then all PET camera models will meet the criteria, and all PET centers will be able to participate in the multicenter study from the viewpoint of PET camera performance. However, the quality of the PET images may not be high enough to demonstrate the efficacy of a new PET drug.

It is desirable that the proposed criteria should conform to the image quality level at which multicenter clinical studies have been performed in order to obtain evidence of the efficacy of a PET drug or to build databases in the academic community, such as ADNI, ADNI2, J-ADNI, and clinical trials of ^18^F-florbetapir and ^18^F-flutemetamol. Therefore, phantom experiments were carried out to obtain the parameter values on the PET camera models that were used in the multicenter clinical studies under the scanning conditions specified for each camera. In such well-organized multicenter studies, the designated PET QC manager examines and qualifies the PET camera of each participating center with the phantoms by determining appropriate reconstruction conditions. Therefore, the scanning details adopted in such studies would provide credible information about the level of image quality that the PET images acquired with each PET drug should satisfy in general. One of the primary limitations of this method is that the gray-white ratios that are central to amyloid analysis are not well or directly tested with the Hoffman phantom. The Hoffman phantom may have applicable complexity in terms of anatomy, but not in terms of distributions, especially for amyloid PET scans. The distributions of the Hoffman phantom are not directly applicable to the method, in particular, the cerebellum and pons typically used as reference tissues.

It is also desirable that most of the currently used PET cameras should be able to meet the phantom test criteria under appropriate scanning conditions so that most PET centers can participate in a multicenter study that adopts the criteria. Therefore, the phantom experiments were also carried out on most PET camera models used in Japan to confirm that the criteria are achievable by selecting appropriate reconstruction conditions for most of them (Table 4). Naturally, the reference values in the criteria might change in the future according to the development and advent of new PET cameras with higher physical performance when older cameras are replaced by newer models.

To optimize the PET image quality between PET centers and between PET cameras in a multicenter study, the investigator is supposed to find such appropriate reconstruction parameters that will generate phantom images satisfying the criteria. The scan time (data acquisition time) may be adjusted depending on the sensitivity of the PET camera so that sufficient gamma ray counts are collected. For PET cameras with poor intrinsic performance, it may be difficult to find reconstruction parameters that will satisfy both resolution (%contrast) and noise, as depicted in Fig. 3, due to a trade-off between image resolution and noise. In this particular case, lengthening the scan time may suppress the noise without degrading the contrast. However, since Fig. 3 represents a case for ^18^F-flutemetamol, in which the standard clinical scan time is as long as 30 min, it may be practically difficult to make it any longer. If no reconstruction parameters or scan time are found for a certain PET camera that can generate phantom images satisfying the criteria, then the investigator may decide not to use the PET camera for a multicenter project. It is, of course, up to the investigator whether to conform strictly to the criteria or to allow some deviation.

As an ^18^F-labeled amyloid PET drug, ^18^F-florbetaben has also been developed and is commercially available in the USA, South Korea, and Europe [19]. The distribution of ^18^F-florbetaben in the brain is similar to ^18^F-florbetapir [39]; however, a difference between ^18^F-florbetapir and ^18^F-florbetaben, which is a nitrogen atom in the chemical structure, affects the pharmacokinetic properties and retention of the tracer in the target region. The imaging window for ^18^F-florbetaben providing the highest contrast between gray matter and white matter begins at 90 min post-injection. According to the sponsor company (Piramal Imaging), the standard injection activity, accumulation time, and scan time for ^18^F-florbetaben are 300 MBq, 90 min accumulation, and 20 min scan, respectively, and the average % brain uptake at 90 min post-injection was 3.5 %ID, based on the data of clinical trials in Japan, leading to the estimated brain activity of 6 MBq at scan start [26]. Therefore, the phantom data interval to be extracted for list mode data acquisition is 355 s for the Hoffman phantom (20 MBq at start) and 180 s for the cylindrical phantom (40 MBq at start) under standard scanning conditions.

## Conclusions

Based on these considerations, we have proposed phantom criteria that will guarantee sufficient quality for multicenter brain PET studies and that can be met by most currently used cameras. The proposed criteria will help raise the quality of multicenter brain PET studies.
